# Species-level characterization of saliva and dental plaque microbiota reveals putative bacterial and functional biomarkers of periodontal diseases in dogs

**DOI:** 10.1093/femsec/fiae082

**Published:** 2024-05-23

**Authors:** Giulia Alessandri, Federico Fontana, Leonardo Mancabelli, Chiara Tarracchini, Gabriele Andrea Lugli, Chiara Argentini, Giulia Longhi, Sonia Mirjam Rizzo, Laura Maria Vergna, Rosaria Anzalone, Alice Viappiani, Francesca Turroni, Maria Cristina Ossiprandi, Christian Milani, Marco Ventura

**Affiliations:** Laboratory of Probiogenomics, Department of Chemistry, Life Sciences, and Environmental Sustainability, University of Parma, Parco Area delle Scienze 11a, 43124 Parma, Italy; Laboratory of Probiogenomics, Department of Chemistry, Life Sciences, and Environmental Sustainability, University of Parma, Parco Area delle Scienze 11a, 43124 Parma, Italy; Parco Area delle Scienze 11a, 43124 Parma, Italy; Department of Medicine and Surgery, University of Parma, Via Volturno 39, 43125 Parma, Italy; Microbiome Research Hub, University of Parma, Parco Area delle Scienze 11a, 43124 Parma, Italy; Laboratory of Probiogenomics, Department of Chemistry, Life Sciences, and Environmental Sustainability, University of Parma, Parco Area delle Scienze 11a, 43124 Parma, Italy; Laboratory of Probiogenomics, Department of Chemistry, Life Sciences, and Environmental Sustainability, University of Parma, Parco Area delle Scienze 11a, 43124 Parma, Italy; Laboratory of Probiogenomics, Department of Chemistry, Life Sciences, and Environmental Sustainability, University of Parma, Parco Area delle Scienze 11a, 43124 Parma, Italy; Laboratory of Probiogenomics, Department of Chemistry, Life Sciences, and Environmental Sustainability, University of Parma, Parco Area delle Scienze 11a, 43124 Parma, Italy; Parco Area delle Scienze 11a, 43124 Parma, Italy; Laboratory of Probiogenomics, Department of Chemistry, Life Sciences, and Environmental Sustainability, University of Parma, Parco Area delle Scienze 11a, 43124 Parma, Italy; Laboratory of Probiogenomics, Department of Chemistry, Life Sciences, and Environmental Sustainability, University of Parma, Parco Area delle Scienze 11a, 43124 Parma, Italy; Parco Area delle Scienze 11a, 43124 Parma, Italy; Parco Area delle Scienze 11a, 43124 Parma, Italy; Laboratory of Probiogenomics, Department of Chemistry, Life Sciences, and Environmental Sustainability, University of Parma, Parco Area delle Scienze 11a, 43124 Parma, Italy; Microbiome Research Hub, University of Parma, Parco Area delle Scienze 11a, 43124 Parma, Italy; Microbiome Research Hub, University of Parma, Parco Area delle Scienze 11a, 43124 Parma, Italy; Department of Veterinary Medical Science, University of Parma, Via Del Taglio 10, 43126 Parma, Italy; Laboratory of Probiogenomics, Department of Chemistry, Life Sciences, and Environmental Sustainability, University of Parma, Parco Area delle Scienze 11a, 43124 Parma, Italy; Microbiome Research Hub, University of Parma, Parco Area delle Scienze 11a, 43124 Parma, Italy; Laboratory of Probiogenomics, Department of Chemistry, Life Sciences, and Environmental Sustainability, University of Parma, Parco Area delle Scienze 11a, 43124 Parma, Italy; Microbiome Research Hub, University of Parma, Parco Area delle Scienze 11a, 43124 Parma, Italy

**Keywords:** canine, chronic gingival inflammation, metagenomics, oral cavity, periodontitis, premolars, shallow shotgun

## Abstract

Periodontal diseases are among the most common bacterial-related pathologies affecting the oral cavity of dogs. Nevertheless, the canine oral ecosystem and its correlations with oral disease development are still far from being fully characterized. In this study, the species-level taxonomic composition of saliva and dental plaque microbiota of 30 healthy dogs was investigated through a shallow shotgun metagenomics approach. The obtained data allowed not only to define the most abundant and prevalent bacterial species of the oral microbiota in healthy dogs, including members of the genera *Corynebacterium* and *Porphyromonas*, but also to identify the presence of distinct compositional motifs in the two oral microniches as well as taxonomical differences between dental plaques collected from anterior and posterior teeth. Subsequently, the salivary and dental plaque microbiota of 18 dogs affected by chronic gingival inflammation and 18 dogs with periodontitis were compared to those obtained from the healthy dogs. This analysis allowed the identification of bacterial and metabolic biomarkers correlated with a specific clinical status, including members of the genera *Porphyromonas* and *Fusobacterium* as microbial biomarkers of a healthy and diseased oral status, respectively, and genes predicted to encode for metabolites with anti-inflammatory properties as metabolic biomarkers of a healthy status.

## Teaser

Metagenomic dissection of the canine oral microbiota to identify bacterial and functional biomarkers of periodontitis and chronic gingival inflammation.

## Introduction

The oral cavity of mammals represents an extremely heterogeneous ecological niche due to its multiple distinct microenvironments, such as teeth, gingival sulcus, tongue, cheek, and hard and soft palate (Kilian et al. 2016, Lamont et al. 2018, Zhang et al. 2018, Ruparell et al. 2020a). For such reason, the bacterial ecosystem inhabiting the oral cavity, generally known as oral microbiota, is considered, together with the gut microbiota, as one of the most complex and dynamic microbial communities of the mammalian body (Lamont et al. 2018, Zhang et al. 2018). Millions of years of coevolution between this indigenous microbial community and its host have led to the establishment and consequent consolidation of various trophic interactions that ultimately influence the oral and systemic well-being of the host (Kilian et al. 2016, Graves et al. 2019, Bell et al. 2020). In this context, the oral microbial ecosystem not only acts as a protective barrier competing with exogenous pathogens and supporting the oral tissue integrity, but it is also involved in the stimulation/regulation of the host mucosa and immune system (Kilian et al. 2016, Lamont et al. 2018). However, beyond these beneficial activities, the oral microbial ecosystem also plays a relevant role in the onset of various oral diseases, including gingivitis, periodontitis, and dental caries (Costalonga and Herzberg 2014, Yamashita and Takeshita 2017, Zhang et al. 2018, Bell et al. 2020).

An alteration of the oral ecosystem equilibrium may rapidly evolve to a dysbiosis status, generating a favorable environment for pathogen colonization and proliferation with a subsequent stimulation and exacerbation of the host immune response contributing to chronic inflammation (Wallis et al. 2015, Bell et al. 2020, Santibanez et al. 2021).

Specifically, the development of periodontal diseases is primarily caused by bacterial plaque accumulation on the periodontium through biofilm development (Dewhirst et al. 2012, Davis et al. 2013, Graves et al. 2019, Ruparell et al. 2020a, 2021, Oba et al. 2021a). As the biofilm thickens through bacterial adhesion, the dental plaque extends into the subgingival sulcus with subsequent oxygen depletion promoting anaerobic bacterial proliferation (Oba et al. 2021a). At this stage, the calcification of dental plaque may occur due to the exchange of calcium and phosphate ions in saliva leading to the accumulation of additional plaque and, consequently, further irritation of gingival tissues (Logan 2006, Carreira et al. 2015, Oba et al. 2021a). Finally, the persistence/continuous growth of supragingival and/or subgingival plaque can cause inflammation of the adjacent gingival tissues soliciting inflammatory response cascade (Oba et al. 2021a,b).

Gingivitis and periodontitis represent the most common oral diseases in dogs, affecting up to 70% of canine patients, a percentage that is reported to dramatically increase for extrasmall and small dog breeds or with increasing age (Hoffmann TaG 1996, Kortegaard et al. 2008, Wallis and Holcombe 2020, Oba et al. 2021a,b, Wallis et al. 2021a,b). Because of its role in periodontal diseases, the assessment of the microbial community of saliva and dental plaque in dogs is of fundamental importance. In this context, the growing concern for the health of this companion animal has prompted the scientific community to characterize the canine oral microbiota (Ruparell et al. 2020a, Oba et al. 2021a). However, despite the latter has been largely studied through amplicon-based next-generation sequencing techniques and culture-dependent approaches (Davis et al. 2013, Wallis et al. 2015, Flancman et al. 2018, Ozavci et al. 2019, Ruparell et al. 2020a, Oba et al. 2021a, Wallis et al. 2021a), an accurate and precise dissection of the canine oral microbiota composition down to the species level as well as the identification of taxonomic and functional microbial biomarkers related to periodontal pathologies are still far from being fully dissected.

In the current study, the species-level taxonomic composition of the oral microbiota of a total of 66 dogs, divided into 30 healthy dogs, 18 dogs with chronic gingival inflammation (CGI), and 18 dogs with periodontitis, was assessed through a shallow shotgun sequencing approach. Specifically, for each enrolled dog, a saliva sample, as well as a dental plaque sample from both an anterior and posterior tooth of the same individual were collected. The obtained data were subsequently used to explore the oral bacterial composition of dogs, identifying the most prevalent and abundant species of the saliva and dental plaque microniches as well as possible taxonomic differences related to the location of dental plaques. Furthermore, possible taxonomic and functional differences were investigated in correlation with health and disease to identify any bacterial biomarker for CGI and/or periodontitis.

## Materials and methods

### Ethical statement

The study protocol was approved by the “Comitato Etico per la Sperimentazione Animale” (CESA) of the University of Parma (reference number: PROT. N. 22/CESA/2021) and conducted in compliance with the rules, regulations, and recommendations of the Ethical Committee of the University of Parma. All animal procedures were carried out in accordance with national guidelines (Decreto Legislativo 26/2014). Furthermore, signed informed consent was obtained from the owners of each dog involved in this study.

### Sample collection

For the purpose of this study, a total of 66 dogs were enrolled, divided into 30 healthy dogs together with 36 dogs equally divided into dogs affected by CGI and periodontitis, respectively (Table S1). All samples were collected within 2 months thanks to the collaboration with three veterinary clinics located in the North of Italy. For each subject, a dental plaque sample was collected from maxillary teeth, including both anterior (tooth position C or I3) and posterior (tooth position P4 or M1) tooth together with a salivary sample (Table S1). Specifically, when sufficient material could not be collected from anterior tooth C or posterior tooth P4, dental plaques were harvested from the adjacent teeth. Furthermore, while dental plaque samples were manually collected through a dental prophylaxis during a routine veterinary visit, saliva was retrieved by rolling a sterile swab under the tongue of each dog for at least 30 s. In detail, to determine the clinical status of each dog, a gingivitis score between 0 and 4 was recorded for each tooth using a combination of the gingival index and sulcus bleeding index, by evaluating probing depth, gingival recession, and furcation exposure, as previously described (Lobprise 2019, Ruparell et al. 2021, Wallis et al. 2021a).

Once collected, dental plaque and saliva were separately conserved into dedicated sterile tubes containing 1 ml and 3 ml of phosphate buffered saline, respectively, kept on ice and shipped to the laboratory under frozen conditions where they were preserved at −20°C until they were processed. Specifically, since different taxonomic compositions characterize the various stages of dental plaque development, to be comparable, only mature dental plaque samples were collected (Holcombe et al. 2014, Jiang et al. 2021). In detail, since early dental plaque turns into mature biofilms within 15/20 days, to collect only mature dental plaque, the latter were collected from dogs that had not undergone a veterinary dental hygiene procedure for at least 2 months (Herrmann et al. 2020). Furthermore, to be involved in this study, dogs had to be free from any pathology except for the ones under examination, and not having undergone treatment with any probiotics, antibiotics, or other drugs during the month prior sample collections. Furthermore, for each dog, age, sex, breed, weight, diet, and possible use of dental chews or medical devices for canine dental cleaning were annotated (Table S1).

### DNA extraction and Illumina shallow shotgun sequencing

DNA extraction from dental plaque samples was performed using the ZymoBIOMICS DNA Miniprep Kit (Zymo Research Corporation, USA), following the manufacturer’s instructions.

Subsequently, the extracted DNA was prepared using the Illumina NexteraXT DNA Library Preparation Kit and following the Illumina NexteraXT protocol. Briefly, DNA samples were enzymatically fragmented, barcoded, and purified encompassing magnetic beads. Furthermore, samples were quantified using the fluorometric Qubit quantification system (Life Technologies, USA), loaded on a 2200 Tape Station Instrument (Agilent Technologies, USA) and normalized to 4 nM. A paired-end sequencing was performed using an Illumina MiSeq sequencer with MiSeq Reagent Kit v3 for 600 cycles (Illumina Inc., San Diego, USA).

### Shallow shotgun sequencing analysis

The obtained .fastq files were filtered to remove reads with a quality of <25 and sequences of *Canis lupus familiaris* DNA, while reads with a length of >149 bp were retained. Specifically, to remove reads with high sequence identity to the *C. lupus familiaris* DNA, the obtained reads were compared to a dog-based genomic database through a METAnnotatorX2 pipeline function (Milani et al. 2021). Quality-filtered data were used for taxonomic profile reconstruction by using the METAnnotatorX2 pipeline, as previously described (Milani et al. 2021). Specifically, when the software fails to assign a sequence correctly and accurately to an already isolated and characterized species, the sequence is classified only down to the genus level (Milani et al. 2021). For each downstream analysis, only classified bacterial-related reads were considered (Table S2). Functional profiling of the sequenced reads was performed with the METAnnotatorX2 bioinformatic platform (Milani et al. 2018, 2021). Functional classification of reads was performed to reveal metabolic pathways based on the MetaCyc database (release 24.1) (Caspi et al. 2016) through RAPSearch2 software (Ye et al. 2011, Zhao et al. 2012).

### Statistical analysis

Origin 2021 Pro (https://www.originlab.com/2021) and SPSS software (www.ibm.com/software/it/analytics/spss/) were used to compute statistical analyses. In detail, pairwise Kruskal–Wallis test analyses tested differences in alpha diversity that is calculated through species richness and Shannon index. Moreover, similarities between samples (beta-diversity) were calculated by Bray–Curtis dissimilarity matrix based on species abundance. Beta-diversity was represented through PCoA using the function “ape” of the R suite package. Furthermore, the fitting analysis was performed through “envfit” function in RStudio and results were plotted on a PCoA using the “plot” function in RStudio. Specifically, the “envfit” function calculated the fitting score for each tested environmental variable, and the empirical *P*-value was corrected through the Bonferroni test. The function “silhouette” in RStudio was used to perform the Silhouette analysis and the functions “hclust” and “cutree” in RStudio were used to calculate the hierarchical clustering of samples, both based on species-level bacterial composition and in particular Bray–Curtis dissimilarity matrix. Moreover, the lme4 mixed linear model (10.18637/jss.v067.i01), which included age, gender, breed, diet, dental cleaning and type of sample as fixed variable, along with clinical status as a random effect, was calculated on Shannon index biodiversity through “lmer” function in RStudio. Furthermore, a specific RDA based on the species-level taxonomic profiles, followed by an ANOVA permutation test, was performed using the “rda” function from the “vegan” package in RStudio.

SPSS software was also exploited for the calculation of the nonparametric test for two independent samples, i.e. the Mann–Whitney U-test, and the nonparametric test Kruskal–Wallis. Specifically, the Mann–Whitney U-test results were subjected to correction for multiple comparisons using the FDR through the EdgeR package. Furthermore, for Kruskal–Wallis statistic, the Bonferroni *post hoc* test was calculated.

### Data deposition

Raw shallow shotgun sequencing data are accessible through SRA under BioProject number PRJNA863598.

## Results and discussion

### Taxonomic classification of the oral bacterial community in dogs

To explore the species-level taxonomical composition of the oral microbiota of dogs, a total of 66 dogs were enrolled (Table S1). For each dog, a saliva sample and two dental plaque samples, obtained from both an anterior and posterior tooth, were collected (Table S1). Furthermore, to investigate possible differences in taxonomic composition and functional potential of these two oral microniches in case of oral diseases, samples were collected from healthy dogs (Alessandri et al. 2020) as well as from dogs affected by one of the two major canine oral diseases, i.e. CGI (18 dogs) or periodontitis (18 dogs) (Table S1). Bacterial DNA extracted from each sample was submitted to shallow shotgun sequencing and data obtained were analyzed through the METAnnotatorX2 pipeline (Milani et al. 2021, Lugli and Ventura 2022). Illumina sequencing generated a total of 7 282 980 filtered reads with an average of 36 782 classified reads per sample, ranging from a minimum of 10 976 to a maximum of 98 200 reads (Table S2). Thus, an adequate number of reads, i.e. at least 10 000, was reached to predict reliable taxonomic profiles from each sample, through the METAnnotatorX2 software, as previously described (Milani et al. 2021). Details regarding the taxonomic classification at the phylum- and genus-level are reported in the Supplementary text (Tables S3 and S4).

**Figure 1.**
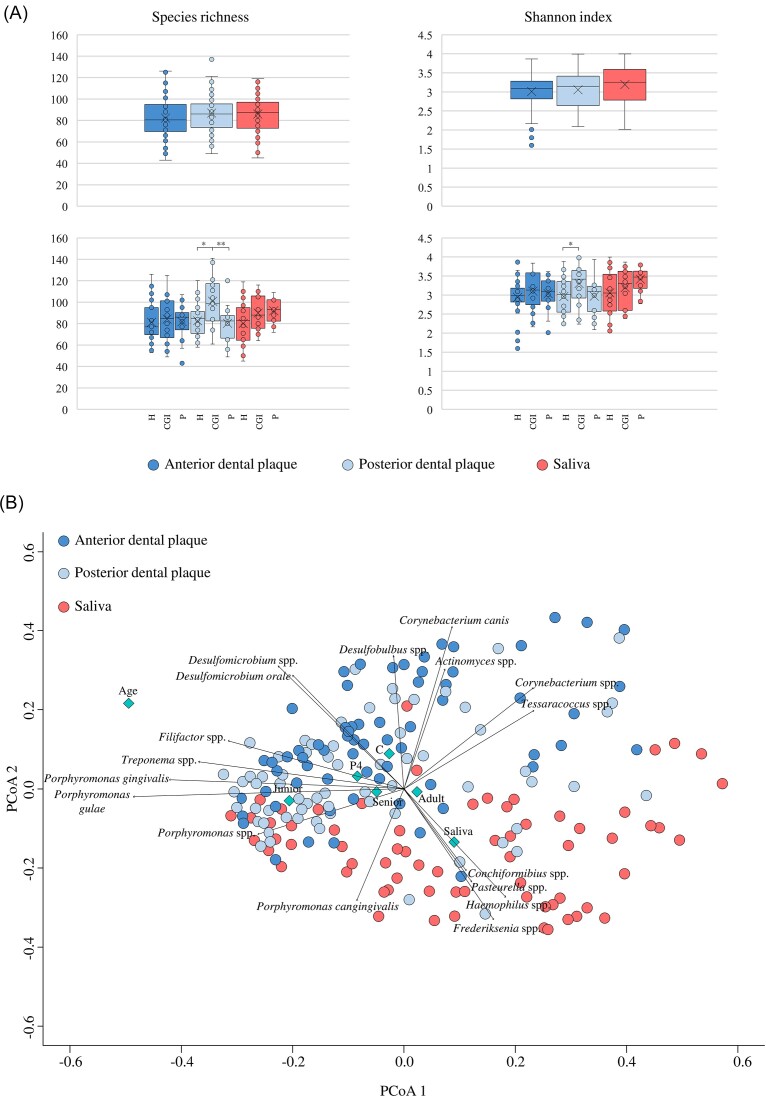
Evaluation of the bacterial diversity of saliva and dental plaque samples in dogs. Panel A reports the box and whisker plots of the calculated species richness (on the left) and Shannon index (on the right) based on the number of bacterial species observed in the three sample types (on the top) and by dividing sample types according to the clinical status (on the bottom). For each plot, the *x*-axis reports the different considered groups (H, healthy; CGI, chronic gingival inflammation; and P, periodontitis), while the *y*-axis shows the number of species. Boxes are determined by the 25th and 75th percentiles. The whiskers are determined by the maximum and minimum values and correspond to the box extreme values. Lines inside the boxes represent the average, while crosses correspond to the median. Panel B shows the two-dimensional Bray–Curtis dissimilarity matrix-based PCoA. In the graph, only those bacterial species with a significant *P*-value and an average relative abundance > 0.5% were reported. In addition, only those metadata that significantly explain the biodiversity of samples were displayed through rhombus.

The generated species-level taxonomic profiles were used to explore the α-diversity among samples. In detail, no significant differences were observed in bacterial complexity among the various types of samples, i.e. saliva or dental plaque from canines or molars, neither through the species richness nor the Shannon index analysis (Kruskal–Wallis test *P*-value > .05 for both statistics) (Fig. 1A), suggesting a similar bacterial complexity among the considered oral microniches. At the same time, no differences in microbial richness were recorded by separating samples based on the clinical condition within each sample type (Kruskal–Wallis test *P*-value > .05), except for dental plaques collected from the molars (Fig. 1A). Indeed, a significant increase in microbial complexity was recorded in the posterior dental plaques of dogs affected by CGI when compared to the healthy ones for both species richness (Kruskal–Wallis test *P*-value = .019) and Shannon index analysis (Kruskal–Wallis test *P*-value = .041) (Fig. 1A). Furthermore, the species richness analysis also showed a significant increment of the microbial complexity in the biofilms collected from the molars of CGI-affected dogs with respect to the dogs with periodontitis (Kruskal–Wallis test *P*-value = .0015) (Fig. 1A). Therefore, overall, α-diversity results indicate that, in general, microbial complexity does not vary among oral ecological microniches nor among different clinical conditions, except for CGI that seems to be associated with a bacterial complexity increment in the dental plaque of posterior teeth in dogs.

In addition, the obtained species-level taxonomic profiles were further used to perform a β-diversity analysis, represented through a principal coordinate analysis (PCoA) based on Bray–Curtis dissimilarity matrix, to evaluate the microbial biodiversity of each canine oral microbiota sample (Fig. 1B and Table S1). Furthermore, to assess whether and which factors may play a role in influencing the bacterial biodiversity of the canine oral microniches, the numerous collected metadata, including age, gender, breed, diet, dental cleaning, type of sample, and clinical status, together with the detected bacterial species, were plotted and integrated into the PCoA using an environmental fitting analysis (Fig. 1B and Table S1). The latter highlighted a statistically significant difference in the taxonomic composition of saliva and dental plaque samples, suggesting that the two different ecological microniches may be characterized by a different microbial community (envfit *R*^2^ = 0.224 and Bonferroni *post hoc* test *P*-value = .003) (Fig. 1B and Table S5). Specifically, while saliva samples strictly correlated with various bacterial species, including *Conchiformibius* spp., *Haemophilus* spp., *Frederiksenia* spp., *Pasteurella* spp., *Porphyromonas cangingivalis*, dental plaque samples resulted to be characterized by *Actinomyces* spp., *Corynebacterium canis, Corynebacterium* spp., *Desulfomicorbium orale, Desulfomicrobium* spp., *Desulfobulbus* spp., *Filifactor* spp., *Porphyromonas gulae, Porphyromonas gingivalis, Porphyromonas* spp., *Tessaracoccus* spp., and *Treponema* spp. (Fig. 1B and Table S5). An observation that was also confirmed by the different prevalence and/or average relative abundance of these bacterial species between saliva and dental plaque samples (Table S6).

Conversely, the different clinical statuses did not significantly impact on the bacterial composition (envfit *R*^2^ = 0.0081 and Bonferroni *post hoc* test *P*-value = .754) (Fig. 1B and Table S5). Probably, the presence of CGI or periodontitis does not cause a drastic alteration of the taxonomic composition of the oral microniches, but rather a modulation of only certain specific microbial biomarkers. Similarly, diet, sex, and dental cleaning did not appear to play a role in influencing the taxonomic composition of saliva and dental plaque in dogs (Fig. 1B and Table S5). However, as only two dogs were on a diet based on bones and raw food (Table S1), this observation could be biased due to the small number of samples.

The only two factors that seemed to affect the oral microbiota of saliva and dental plaque microniches in dogs were represented by breed and age (Fig. 1B and Table S5). In detail, to assess the impact of age on the biodiversity of the oral microbiota in dogs, the collected samples were divided into three different age groups, i.e. junior (9–24 months), adult (25–96 months), and senior (>97 months), as previously described (Alessandri et al. 2019). These two determinants have been already indicated as main variables influencing the onset of oral pathologies in dogs with a preponderance of cases for small dog breeds and/or with increasing age (Oba et al. 2021b, Wallis et al. 2021a,b). Probably, the physiological and structural differences of the oral cavity among dog breeds may play a role in modulating the taxonomic composition of saliva and dental plaque microbiota leading to a different susceptibility to the onset of oral pathologies based on breed.

Overall, these observations highlighted that certain bacterial species are typical of either dental plaques or saliva, suggesting clear differences in the bacterial composition of these two oral microniches, regardless of the clinical status, and that the taxonomic composition of the oral microbiota of dogs is not influenced by factors other than breed and age.

Furthermore, to identify the potential impact of considered variables on biodiversity, as measured by the Shannon index, a mixed linear model approach was employed. Specifically, the lme4 mixed linear model (Bates et al. 2015), which included age, gender, breed, diet, dental cleaning, and type of sample as fixed variable, along with clinical status as a random effect, revealed a statistically significant effect of certain fixed variables on Shannon index biodiversity. Specifically, only those interactions that showed either a *t*-value >2 or ←2 were considered as statistically significant. Based on these cut-offs, among all evaluated multiple-way interactions, only dental cleaning as well as the 2-way interaction involving gender and dental cleaning as variables appeared to have a significant effect on microbial biodiversity (*t*-value of −2.516 and 2.729, respectively). These results were further investigated through a Redundancy Analysis (RDA) based on the species-level taxonomic profiles, followed by an ANOVA permutation test. In detail, with an ANOVA *P*-value of .002 and an explained variance corresponding to 5.08% for the model incorporating age, gender, breed, diet, dental cleaning, and type of sample independent variables, this analysis suggests that, although the considered variables may partially explain the observed diversity in taxonomic profiles, their impact is very limited. Moreover, an additional RDA analysis including only gender and dental cleaning metadata resulted in a *P*-value of .378 that, exceeding the conventional significance level of .05, suggests that the differences observed in the data could likely be attributed to chance rather than to a systematic effect of the studied independent variables on the taxonomic composition of samples. Therefore, overall, these analyses indicate that, while an impact on the Shannon biodiversity was observed by the lme4 analysis, their combined impact on the variability of the taxonomic profiles is not significant.

In addition, beyond the assessment of α- and β-diversity, to further identify possible specific bacterial biomarkers correlated with CGI and/or periodontitis, a taxonomic insight into the saliva and dental plaque microbiota of healthy dogs was first assessed, followed by the identification of possible bacterial and functional biomarkers for CGI and periodontitis.

### Uncovering the salivary microbiota of healthy dogs

**Figure 2.**
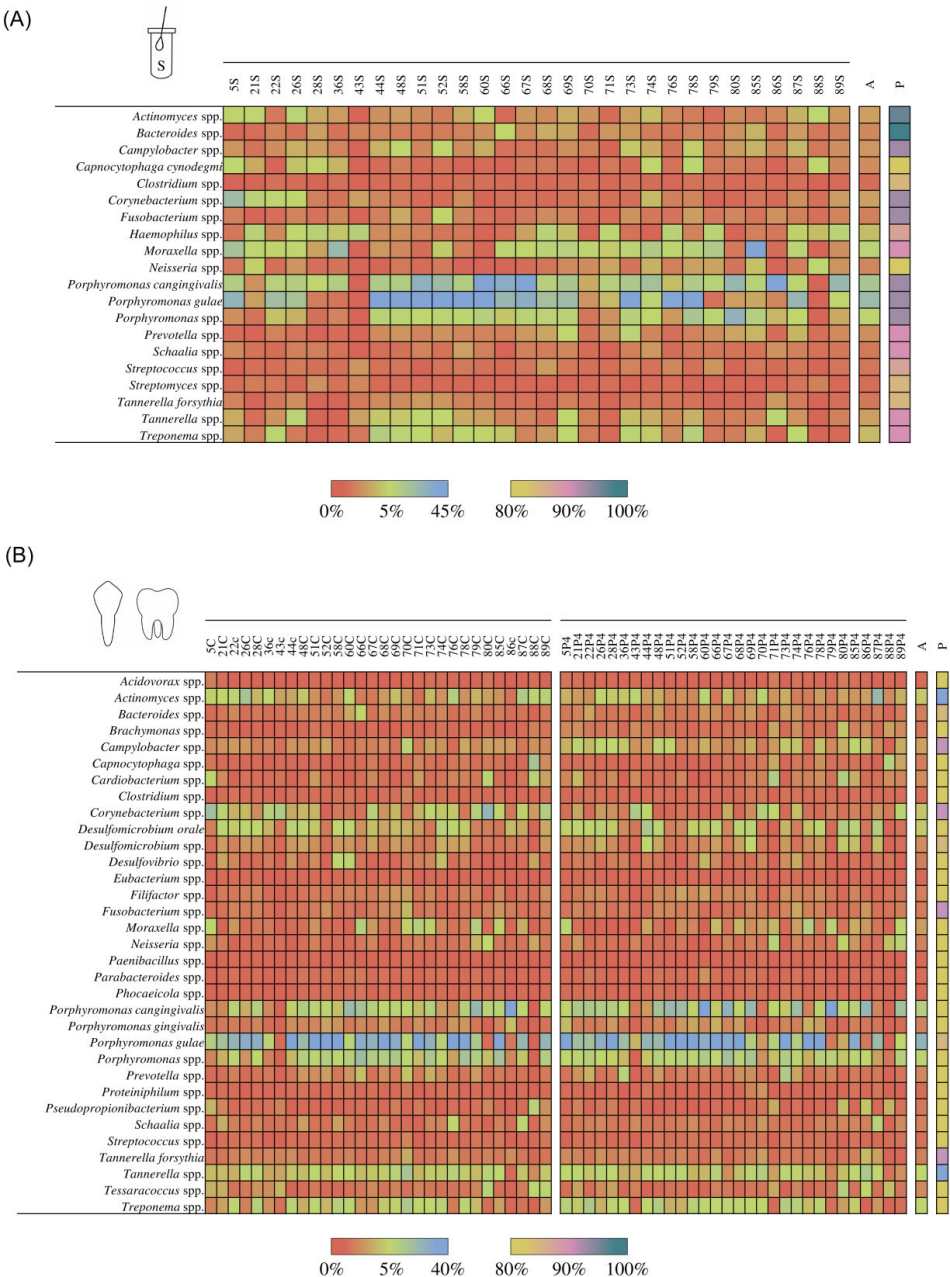
Most prevalent bacterial species in the salivary and dental plaque microbiota of healthy dogs. Panels display the average relative abundance of the most prevalent species (prevalence >80%) for each saliva (panel A) and dental plaque samples (panel B). The column on the left reports the bacterial species, while each column of the heat map displays the relative abundance of each bacterial species per each sample reported on the top of the graph. Columns on the right of each heat map show the average relative abundance (A) and prevalence (P) of each bacterial species.

To explore the species-level bacterial composition of the saliva microbiota in dogs, the taxonomic profiles of the 30 salivary samples collected from healthy dogs, established through the METAnnotatorX2 software, were exploited. In detail, to identify the most representative bacterial species of the canine salivary microbiota, only those bacterial taxa with a prevalence >80% were considered, as previously described (Alessandri et al. 2019, 2020). Based on this criterion, 20 bacterial species were identified as highly conserved taxa in the saliva microbiota of healthy dogs (Fig. 2A). Among the latter, the two most abundant species belonged to the genus *Porphyromonas*, including *P. gulae* (average relative abundance of 15.62%) and *P. cangingivalis* (10.87%) (Fig. 2A). Notably, members of this bacterial genus are widely reported as predominant commensals of the oral cavity of healthy dogs (Ruparell et al. 2020a, Oba et al. 2021a,b, 2022, Portilho et al. 2023). Thus, this data strengthens the ecological relevance of *Porphyromonas* species in the salivary microbial ecosystem of dogs. In addition, although with an average relative abundance considerably lower than the *Porphyromonas* species, other two classified taxa, i.e. *Tannerella forsythia* (0.43%) and *Capnocytophaga cynodegmi* (0.86%), were found to be shared by more than 80% of salivary samples from healthy dogs (Fig. 2A). Interestingly, both species are generally described to be part of the oral cavity microbiota in dogs (Oh et al. 2015, Oba et al. 2021a,b, 2022), thus suggesting extensive coevolution between these taxa and the canine oral microbial ecosystem.

However, beyond these classified species, the other most prevalent bacterial taxa of the canine salivary samples matched with unclassifiable species belonging to 16 different bacterial genera (Fig. 2A). Among the latter, the most abundant taxa (average relative abundance >1%) belonged to the genera *Moraxella* (average relative abundance of 4.49%), *Porphyromonas* (3.54%), *Haemophilus* (1.97%), *Treponem*a (1.87%), *Tannerella* (1.34%), *Actinomyces* (1.31%), *Corynebacterium* (1.30%), and *Campylobacter* (1.10%). Of note, all these bacterial genera are commonly detected in the oral cavity of healthy dogs (Oba et al. 2021a,b, Alessandri et al. 2022), thus strengthening the ecological relevance of these taxa in the salivary microbiota of dogs (Fig. 2A). The only exception was represented by the genus *Haemophilus* whose presence has not been detected in previous studies. In this context, considering that the salivary microbiota of dogs seemed to be largely dominated by bacteria not yet identified and that this microbial community is directly involved in influencing the health condition of the host (Oh et al. 2015, Davis and Weese 2022), the application of a culture-based approach is necessary to isolate and characterize these dominant unclassifiable taxa to better understand their role in the assembly and development of the canine salivary microbiota.

### Evaluation of the species-level biodiversity of the dental plaque microbiota of healthy dogs

In addition to saliva, the microbial community involved in the establishment and development of the dental plaque biofilm also plays a crucial role in the onset of pathological conditions, such as CGI or periodontitis (Graves et al. 2019, Ruparell et al. 2020a, 2021). However, to identify possible bacterial biomarkers responsible for oral diseases, it is first necessary to define the species-level taxonomic composition of dental plaque in healthy dogs.

Taxonomic composition reconstruction revealed that the most prevalent (prevalence >80%) and abundant microbial species (average relative abundance >5%) of the canine dental plaques in healthy dogs corresponded to *P. gulae* (average relative abundance of 21.40%), *P. cangingivalis* (7.93%), and *Porphyromonas* spp. (5.26%) (Fig. 2B), thus indicating the predominance of this bacterial genus not only in the canine salivary microbiota, but also in the dental plaque microbial ecosystem. An observation that leads to hypothesize that, probably, both these ecological microniches of the canine oral cavity may act as a reservoir for these species (Fig. 2). However, with 33 bacterial species with a prevalence >80%, the dental plaque microbiota displayed a higher number of highly prevalent species compared to salivary samples. This finding suggests, as already reported for humans (Ren et al. 2017, Li et al. 2022), a larger “core” microbial community of the canine dental plaque microenvironment, when compared to the salivary microbiota. Specifically, beyond members of the genus *Porphyromonas, D. orale* as well as members of the genera *Actinomyces, Campylobacter, Cardiobacterium, Corynebacterium, Desulfomicrobium, Moraxella, Prevotella, Tannerella, Tessaracoccus*, and *Treponema* resulted to be the most abundant with an average relative abundance >1% (Fig. 2B), denoting the ecological adaptation of these species to the canine oral cavity environment. Furthermore, even if not included in the “core” dental canine plaque microbiota, *C. canis* and *Porphyromonas gingivicanis* displayed a high abundance (average relative abundance of 9.29% and 2.82% and prevalence of 78.33% and 73.33%, respectively) in the dental biofilm of dogs. An observation that suggests the ecological relevance of these two taxa, when present, in the assembly of the canine dental plaque microbiota.

Altogether, taxonomic profiles retrieved from saliva and dental plaque samples from healthy dogs evidenced our currently limited knowledge of the host-associated bacterial species inhabiting these ecological niches despite the close relationship between dogs and humans and their putative implications on human health. In this context, the obtained data underlines the urgent need for research efforts aimed at isolating and characterizing this microbial dark matter.

### Location-related differences in the taxonomic composition of dental plaque of healthy dogs

The different pH values as well as oxygen tension and mucosal surface that characterize the different ecological microniches of the canine oral cavity are just some of the main intrinsic factors that, together with external determinants such as exposure to food or mechanical insults, affect the local taxonomic composition of each oral microniche (Ruparell et al. 2020a,b, Oba et al. 2021a,b). Therefore, to evaluate whether also dental plaque localization may have an impact on bacterial biodiversity, taxonomic profiles obtained from dental plaque samples collected from the anterior (canines) and posterior (premolars) teeth of each dog were compared.

Interestingly, the nonparametric Mann–Whitney U-test for two independent samples corrected for multiple comparisons using the false discovery rate (FDR), revealed that three of the taxa previously identified as dominant in the dental plaque microbiota of dogs, i.e. *Campylobacter* spp., *Corynebacterium* spp., and *Brachymonas* spp. exhibited significantly different relative abundances between anterior and posterior teeth, suggesting that the location of the dental plaque biofilm plays a crucial role in influencing the relative abundances of some of the major microbial players of the dental plaque microbiota (Table S7). In detail, *Campylobacter* spp. (average relative abundance of 1.24% and 2.12% in the canine and premolar teeth, respectively) and *Brachymonas* spp. (0.20% and 0.68% in the canine and premolar teeth, respectively) displayed a significant increment in the dental plaque from premolars when compared to those from canines (Table S7). On the other side, the average relative abundance of *Corynebacterium* spp. (3.88% and 1.54% in the canine and premolar teeth, respectively), showed a statistically significant increase in the anterior tooth dental plaque compared to that of premolars (Table S7).

In addition, *Desulfobulbus* spp. and *Pasteurella dagmatis* displayed not only a higher prevalence but also a significant average relative abundance increments in the anterior and posterior dental plaques, respectively (Table S7).

All together, these findings pointed out that biofilm localization, i.e. on anterior or posterior teeth, may have a role in modulating the relative abundances of certain bacterial species that correspond to both “core” and minority bacterial species. Thus, anterior and posterior dental plaques have been considered separately for the subsequent comparison with dogs affected by CGI and periodontitis.

### Identification of community state types in the salivary and dental plaque microbiome of healthy dogs

Although the identification of a “core” microbial ecosystem of the various microniches of the oral microbiota allowed the identification of the most prevalent and abundant bacterial species across a sample cohort, it does not investigate the presence of possible distinct motifs in the overall community composition profiles, thereby ignoring the presence of highly abundant and representative bacterial taxa in a subset of samples (Arumugam et al. 2011, Costea et al. 2018, Alessandri et al. 2022). In this context, to evaluate the possible presence of different compositional patterns, also known as community state types (CSTs), among canine salivary and dental plaque samples of healthy dogs, the taxonomic profiles related to the 30 saliva and 60 dental plaque samples were distinctly used for the calculation of the Silhouette index (Fig. S1), i.e. an unsupervised index, to define the ideal number of clusters to optimally subdivide samples according to their bacterial composition. Subsequently, based on this data, a hierarchical clustering analysis was performed to classify samples into the predicted number of clusters (Fig. S2). However, to be considered as real CSTs, each cluster must include at least three samples.

The prediction of common motifs in the overall community composition profiles from salivary samples, here referred as saliva community state types (s-CSTs), highlighted the separation of samples into two real s-CSTs. Specifically, a main cluster (s-CST3) containing 17 salivary samples was characterized by a high average relative abundance (>10%) of *P. gulae* and *P. cangingivalis*, while s-CST5, counting a total of six salivary samples, resulted to be dominated only by *P. gulae* (Fig. S2). Interestingly, despite the clustering of samples into different groups, the cluster-characterizing taxa corresponded to the most abundant species of the salivary samples of dogs when considered all together, thus confirming the evolutionary adaptation of these species to this specific microniche of the canine oral cavity.

On the other side, the prediction of the canine dental plaque community state types (c-dp-CSTs) allowed the identification of three different c-dp-CSTs (Fig. S2). In depth insights into the latter revealed that most of samples fell within c-dp-CST1 (18 samples), which was dominated by *P. gulae* (average relative abundance of 26.79%), strengthening the central ecological role of this taxon in the microbial ecosystem of the canine oral cavity (Fig. S2). Conversely, while c-dp-CST2 was codominated by *C. canis* and *P. gulae*, c-dp-CST3 was characterized by a predominance of *Corynebacterium* spp. and *Corynebacterium freiburgense* (Fig. S2). Of note, while some cluster-dominant species, including *P. gulae* and *Corynebacterium* spp., corresponded to “core” anterior dental plaque bacterial taxa (Fig. 2), corroborating their role as commensal microorganisms in anterior biofilms, the stratification of anterior dental plaque samples into clusters highlighted the cluster dominance of other two species not previously identified as “core” member of the anterior dental plaques, i.e. *C. canis* and *C. freiburgense* (Fig. S2), thus suggesting the ecological relevance of these species in the assembly and development of the dental plaque microbiota in certain dogs. Not by chance, the latter taxa have been reported as typical microbial colonizers of the canine oral cavity (Funke et al. 2009, 2010, Santibanez et al. 2021, Thongma et al. 2023).

The assessment of the premolar teeth dp-CSTs (p-dp-CSTs) highlighted, instead, the subdivision of samples into four different clusters of which only two represented real CSTs comprising more than three samples (Fig. S2). Specifically, while p-dp-CST1, counting four samples, was dominated by *N. dumasiana* (average relative abundance of 15.97%), the main cluster comprising 22 samples, i.e. p-dp-CST4, showed a predominance of *P. gulae* (29.62%). Furthermore, despite the reduced number of samples, dominant species were also identified for the other two clusters, i.e. *C. canis* for cluster 2 and *P. cangingivalis* together with *P. gingivicanis* for cluster 3 (Fig. S2).

All together these data showed that, beyond the clear dominance of species of the genus *Porphyromonas* both in the saliva and dental plaque microbiota of dogs regardless of location, the species *C. canis* appeared to be a possible dominant taxon only of the dental plaque biofilms, suggesting the latter species as a main colonizer of the dental plaque ecological microniches. Finally, the HCL analysis highlighted that also *N. dumasiana* and *C. freiburgense* may be considered as dominant bacterial players in certain posterior and anterior dental plaque samples, respectively, indicating their ecological relevance in the formation of the dental plaque microbiota in dogs.

#### Prediction of putative CGI and/or periodontitis bacterial biomarkers in the oral microbiota of dogs from different microniches

Reconstruction of a detailed overview of the healthy canine oral microbiota guided us in the assessment of possible microbial biomarkers involved in the onset of CGI and/or periodontitis. For this purpose, saliva and dental plaque samples from dogs with CGI and periodontitis were compared to those collected from healthy dogs by using the nonparametric Kruskal–Wallis test with the Bonferroni correction.

**Figure 3.**
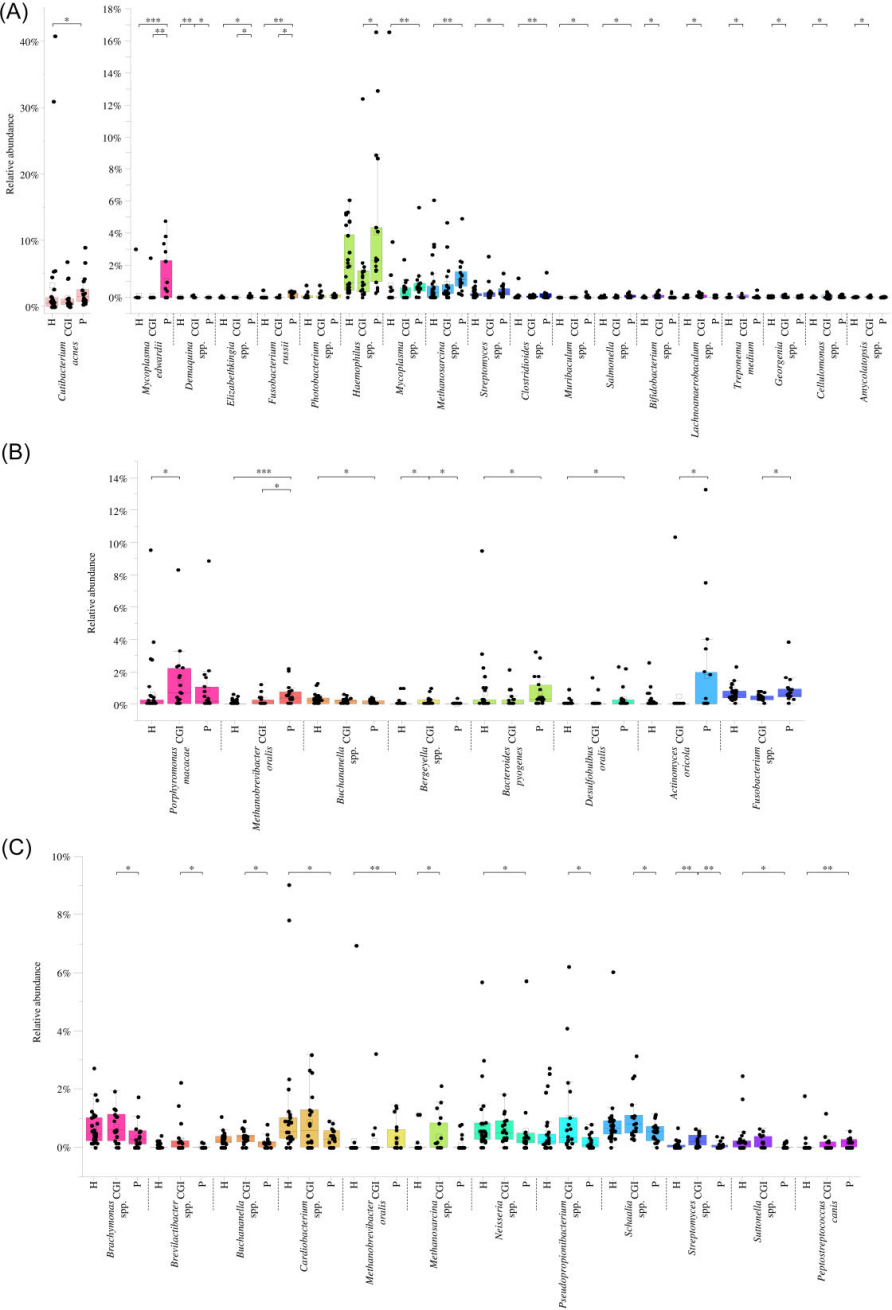
Putative periodontal disease microbial biomarkers. Panels A, B, and C display the box and whisker plots of the relative abundances of those bacterial species that significantly differ among the three clinical conditions in saliva, anterior, and posterior dental plaques, respectively. For the posterior dental plaques, only those species with an average relative abundance >0.2% in at least one clinical status were reported. The *x*-axis reports the bacterial species, while the *y*-axis displays the relative abundance. Boxes are determined by the 25th and 75th percentiles. The whiskers are determined by the maximum and minimum values and correspond to the box extreme values. Lines inside the boxes represent the average, while the square corresponds to the median. H, healthy; CGI, chronic gingival inflammation, and P, periodontitis. *, Krusal–Wallis *P*-value < .05; **, Kruskal–Wallis *P*-value < .01; and Kruskal–Wallis *P*-value < .001.

Remarkably, *Streptomyces* spp. and *Haemophilus* spp., above-identified as “core” bacterial species of the salivary microbiota of dogs (Fig. 2), showed a significantly higher abundance in dogs affected by periodontitis when compared to the healthy and CGI-affected ones, respectively (Fig. 3A and Table S8), suggesting that an increase of this prevalent species may be considered as a biomarker of periodontitis. In contrast, the other 17 bacterial species that significantly differed among the clinical conditions corresponded to minority taxa, pointing at subdominant species as biologically relevant markers in saliva (Fig. 3 and Table S8). In this regard, *Mycoplasma edwardii, Fusobacterium russii*, and *Elizabethkingia* spp. showed a significant increased average relative abundance in salivary samples from dogs with periodontitis when compared to the other two groups (Fig. 3A and Table S8), while the average relative abundances of *Mycoplasma* spp., *Methanosarcina* spp., *Clostridioides spp*., and *Salmonella* spp. resulted to be significantly higher in the saliva of dogs with periodontitis with respect to the healthy ones (Fig. 3A and Table S8). Therefore, the increased abundance of these species may be considered as potential periodontitis salivary bacterial biomarkers. In this context, while *M. edwardii* have been depicted as pathogen of the respiratory tract in dogs (Jambhekar et al. 2019, Alves et al. 2023), various species belonging to the genus *Elizabethkingia* have been recognized as emerging pathogens responsible of various septicemia cases (Bordelo et al. 2016, Zajmi et al. 2022, Weese et al. 2023). At the same time, a member of the genus *Fusobacterium* with a high abundance in the human oral cavity, i.e. *Fusobacterium nucleatum*, and in general other representatives of this taxon, have been shown to produce several virulence factors, including adhesin, endotoxins, and serine proteases that not only allow this species to better survive in a hostile environment, but ultimately trigger the host immune response, causing both local and systemic inflammation (Ding and Tan 2016, de Andrade et al. 2019). Therefore, it is possible to suggest that other less characterized species of the genus *Fusobacterium*, such as *F. russii*, may perform similar features in dogs. Furthermore, *Muribaculum* spp. was not only absent in salivary samples from healthy dogs, but its relative abundance was significantly higher in salivary samples collected from dogs with periodontitis, suggesting that the presence of members of this bacterial genus is closely associated with a periodontitis condition in salivary samples and its presence can be considered as a clear biomarker of this oral disease in dogs (Fig. 3A and Table S8). On the other side, *Bifidobacterium* spp., *Lachnoanaerobaculum* spp., *Cellulomonas* spp., *Georgenia* spp., *Amycolatopsis* spp., and *Treponema medium* displayed a significantly higher average relative abundance in the CGI salivary samples when compared to the healthy ones (Fig. 3A and Table S8), thus electing these six species as microbial biomarker of a CGI condition. Not by chance, *T. medium* has been described as a microorganism involved in the etiology of gingivitis and periodontitis in humans, corroborating the potential role as a CGI biomarker in dogs when examining the salivary microbiota (Zeng et al. 2021, Oba et al. 2022). Overall, this data suggests that oral diseases are associated with a dysbiosis of the saliva microbial community corresponding to an increase of the average relative abundance of certain taxa that are either “core” or “accessory” species in the salivary samples of healthy dogs.

Similarly to saliva, significant changes in the average relative abundances of both highly prevalent and minority species were observed also for dental plaque samples among the three considered clinical conditions (Fig. 3B and C and Table S8), thus suggesting that oral diseases are accompanied by a marked alteration of the oral equilibrium ecosystem not only in saliva but also in dental plaques. Specifically, the biofilms collected from the anterior teeth of dogs suffering from periodontitis displayed a significantly higher abundance of *Actinomyces oricola* and the “core” taxon *Fusobacterium* spp. coupled with *Bacteroides pyogenes* and *Desulfobulbus oralis* when compared to the CGI-affected and healthy ones, respectively (Fig. 3B and Table S8). In addition, *Methanobrevibacter oralis* resulted to be significantly more abundant in the anterior dental plaques of dogs with periodontitis with respect to the other two clinical conditions (Fig. 3B and Table S8). Overall, these results suggest that a high proportion of these taxa can be considered as marker of a periodontitis status in the anterior dental plaque biofilms. Notably, as above reported for saliva, members of the genus *Fusobacterium* are widely recognized as potential pathogen of the oral cavity (Goldstein et al. 2017, Crowley et al. 2024, Krieger et al. 2024), while members of the genus *Actinomyces* have been positively correlated with oral diseases in dogs (Oba et al. 2021), particularly *A. oricola* that was isolated for the first time from a human dental abscess (Hall et al. 2003). Similarly, the methane producer *M. oralis* and the sulphate-reducing *D. oralis* have been strictly associated with severe periodontal disease both in humans and dogs (Cross et al. 2018, Niemiec et al. 2021), while *B. pyogenes* have been frequently described as an animal pathogen (Fernandez Vecilla et al. 2023, Sadhwani et al. 2024). In addition to periodontitis biomarker, *Porphyromonas macacae* and *Bergeyella* spp. were significantly more abundant in CGI-affected dogs when compared to those with periodontitis and the two other two clinical conditions, respectively (Fig. 3B and Table S8), suggesting the high abundance of these two taxa as possible bacterial biomarker of CGI in the anterior dental plaques of dogs. Finally, *Buchananella* spp. was the only bacterial taxon with a significantly higher relative abundance in healthy dogs when compared to those with periodontitis (Fig. 3B and Table S8), indicating that members of this genus may be considered as potential biomarker of a healthy status. However, since very little is known about the genus *Buchananella*, further studies aimed at isolating and characterizing members of this taxon are necessary to dissect their role in the oral cavity microbiota of healthy dogs and to fully understand their potential biological functions.

Contrarily from the anterior dental plaques in which only eight species differed among the clinical conditions, the Kruskal–Wallis statistics applied to dental plaque samples from premolars revealed 39 bacterial taxa with a significantly altered average relative abundance in the case of oral diseases (Fig. 3C and Table S8). Specifically, the relative abundance of 34 of the latter was significantly higher in posterior dental plaque samples collected from dogs suffering from CGI (Fig. 3C and Table S8), suggesting that the investigation of the posterior dental plaque microbiota may be used as an excellent indicator of a CGI status. Among the species with an average relative abundance >0.2% in at least one group, *Brachymonas* spp., *Brevilactibacter* spp., *Buchananella* spp., *Pseudopropionibacterium* spp., and *Schaalia* spp. were significantly higher in CGI when compared to periodontitis (Fig. 3C and Table S8), while *Methanosarcina* spp. and *Streptomyces* spp. showed a significantly higher abundance in dogs with CGI when compared to the healthy ones and both the other two clinical conditions, respectively (Fig. 3C and Table S8). On the other side, the “core” species *Cardiobacterium* spp. coupled with *Neisseria* spp., recorded a significant increment in relative abundance in the biofilms of healthy dogs when compared to those with periodontitis (Fig. 3C and Table S8), suggesting that a high proportion of these two taxa may correlate with a healthy oral cavity when examining the posterior dental plaques. Finally, as also observed for anterior dental plaques, also for the posterior biofilms, the abundance of *M. oralis* resulted to be significantly higher in dogs with periodontitis when compared to the healthy ones (Fig. 3C and Table S8), reinforcing the correlation between this taxon and a periodontitis status when considering dental plaque samples regardless of their location.

Overall, these results not only show that the three different clinical conditions are characterized by significant alterations in the relative abundance of certain bacterial species, but also that these shifts in taxonomic composition in the evolution from a healthy status to periodontitis seem to be specific for each oral ecological microniche.

#### Prediction of oral microbial metabolic pathway signatures differentiating the dental plaque microbiome in healthy or periodontal conditions in dogs

Availability of shallow shotgun metagenomics data allowed also to explore the metabolic potential of the oral microbiota in health and periodontal disease in terms of enzymatic reaction profiles based on the MetaCyc database and the Enzymatic Commission classification.

Interestingly, while in saliva samples the abundance of a total of 172 enzyme-encoding genes significantly differed between healthy and periodontitis-affected dogs, only 41 and 69 enzymes resulted to be differentially present in the oral microbiota between the two clinical conditions from anterior and posterior dental plaque, respectively, as evidenced by the application of the Mann–Whitney U-test with FDR correction (Table S9).

**Figure 4.**
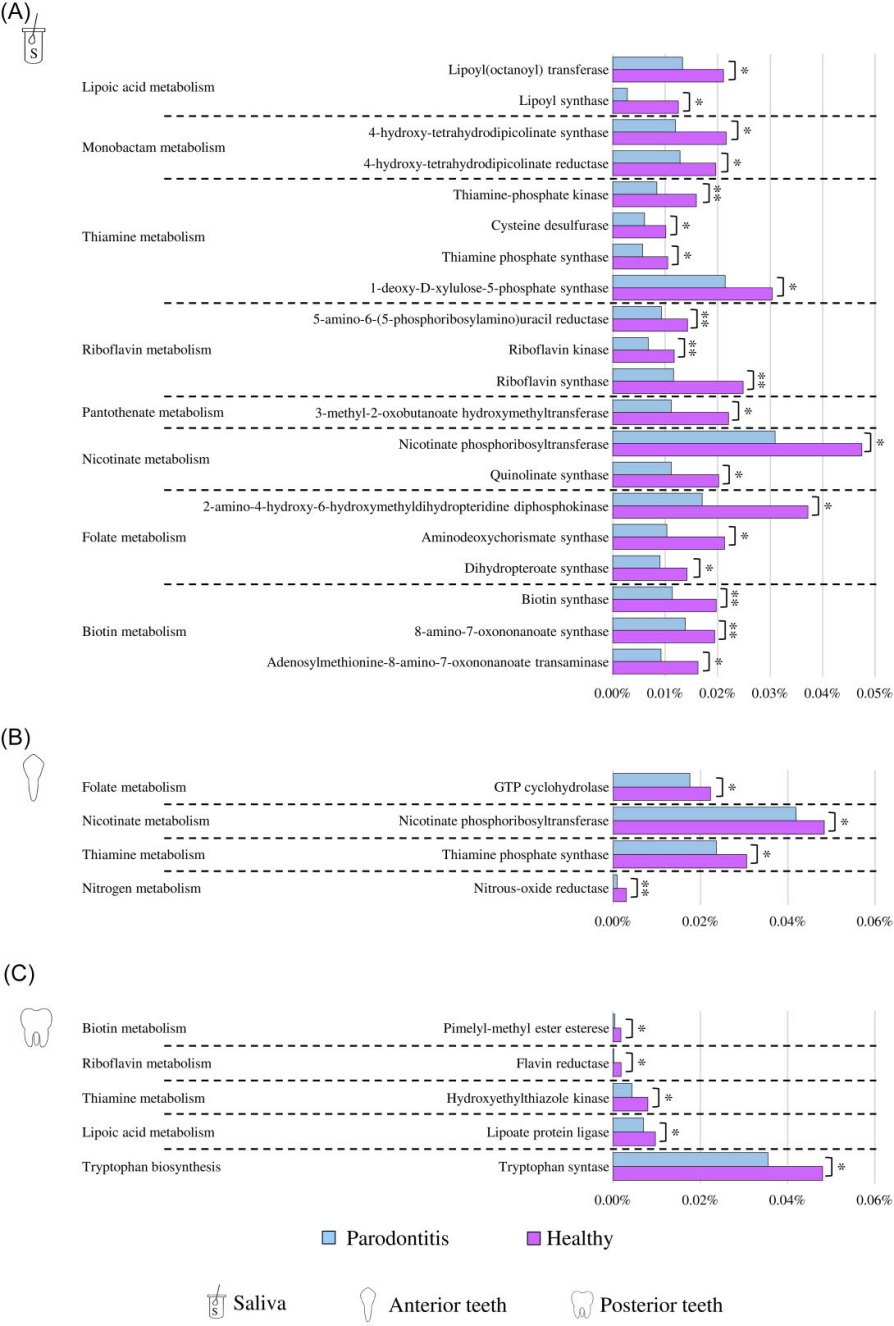
Putative functional biomarkers related to periodontitis. Bar plots indicate the average relative abundance of each enzyme-encoding genes in healthy and periodontitis-affected dogs in saliva (A), anterior (B), and posterior (C) dental plaques. Only enzymes involved in the stimulation/regulation of the host immune system were reported.

Specifically, for all three microniches, among the various enzyme-encoding genes significantly more abundant in the oral microbiota of healthy dogs when compared to that of periodontitis-affected dogs, several genes appeared to be involved in the biosynthetic pathways of various B group vitamins, including biotin (B8), folate (B9), nicotinamide (B3), pantothenate (B5), riboflavin (B2), and thiamine (B1) (Fig. 4 and Table S9). In this context, since B group vitamins are essential micronutrients that participate in a plethora of metabolic, physiological, and regulatory processes comprising immune cell regulation towards an anti-inflammatory condition and suppression of the colonization by pathogenic bacteria in the intestine (Ueland et al. 2017, Peterson et al. 2020, Uebanso et al. 2020), it can be suggested that the increased number of genes involved in B vitamin production may play a similar beneficial role also in the oral cavity of dogs.

Furthermore, in depth insight into the enzymatic reaction profiles from saliva samples highlighted that two genes predicted to encode enzymes involved in the monobactam metabolism, a β-lactam antibiotic with a specific spectrum of activity against Gram-negative aerobes, were significantly enriched in the salivary microbiota of healthy dogs when compared to that from dogs affected by periodontitis (Fig. 4 and Table S9) (Bush and Bradford 2016, De Angelis et al. 2020). In this context, the higher abundance of genes involved in the production of this antibiotic may play a role in modulating the taxonomic composition of the salivary microbiota toward a healthy microbial ecosystem by targeting certain potential pathogens of the canine oral cavity. In addition, a lipoyl(octanoyl) transferase and a lipoyl synthase, two genes predicted to be involved in the lipoic acid biosynthetic pathway, are significantly reduced in the saliva of dogs affected by periodontitis (Fig. 4 and Table S9). In this context, since lipoic acid is an antioxidant that acts by regulating mechanisms of inflammation in several chronic diseases by exerting anti-inflammatory effects (Poles et al. 2021, Sztolsztener et al. 2022), it can be suggested that the increased number of genes involved in lipoic acid production in the genomes of bacteria colonizing the saliva of healthy subjects may also play an anti-inflammatory role in the oral cavity.

The posterior dental plaques of healthy dogs, instead, were characterized by a statistically significant increment of the abundance of a gene predicted to encode a nitrous-oxide reductase, which is a central gene of the nitrate–nitrite–nitric oxide pathway, when compared to those affected by periodontitis (Fig. 4 and Table S9). Nitrate and nitrate-reducing bacteria have been proposed as potential prebiotics and probiotics, respectively, for human oral and systemic health, since the metabolite produced by nitrate reduction, i.e. nitrite, seems to prevent oral disease and improve systemic health (Takahashi 2015, Rosier et al. 2020a,b). Therefore, the significant increase in the abundance of a gene encoding for nitrous-oxide reductase in the dental plaque microbiome of healthy dogs can be considered as a microbial metabolic marker associated with healthy conditions. (Pacher et al. 2007, Sharma et al. 2007, Alves et al. 2021, Leclerc et al. 2021). In parallel, a gene coding a tryptophan synthase was significantly more abundant in the posterior dental plaque biofilm of healthy dogs when compared to those affected by periodontitis (Fig. 4 and Table S9). In this context, since tryptophan has been identified as an essential amino acid able to exert anti-inflammatory effects in the intestine, guaranteeing gut homeostasis, regulating intestinal barrier function, and enhancing intestinal barrier integrity (Gao et al. 2018, Scott et al. 2020, Fiore and Murray 2021), it can be suggested that the increased number of genes involved in tryptophan production in the genomes of bacteria colonizing the dental plaque of healthy subjects may also play an anti-inflammatory role in the oral cavity.

Overall, these data support the notion that periodontitis not only alters the taxonomic composition of the salivary and dental plaque microbiota in dogs, but also the genetic repertoire of this bacterial ecosystem. Indeed, the microbial community of healthy dogs seems to be characterized by a higher number of genes that may exert anti-inflammatory function and limit the proliferation of pathogens when compared to the periodontitis-affected ones. However, although the shallow shotgun approach provides information on the functional potential of the oral microbiota of dogs allowing the assessment of those genes whose abundance differs between clinical conditions and, therefore, the identification of possible functional biomarkers, it does not allow to carry out an accurate and precise gene–bacterial species association, preventing from evaluating whether the genes that significantly differed between clinical conditions belong to the genome of those species above identified as microbial biomarkers (Lugli and Ventura 2022). In this context, the application of a deep shotgun approach is necessary to obtain a more accurate insight into the oral microbiome of dogs allowing a precise and detailed association between functions and bacterial species.

## Conclusions

Dental plaque and saliva microbial ecosystem are involved in the onset of oral pathologies, including CGI and periodontitis (Costalonga and Herzberg 2014, Yamashita and Takeshita 2017, Zhang et al. 2018, Bell et al. 2020). Specifically, the latter are widespread in dogs and, if not properly treated, may have a serious impact on canine health (Oba et al. 2021a,b, Wallis et al. 2021a). In this context, to characterize the saliva and dental plaque microbiota of healthy dogs at the species-level, samples collected from 30 dogs were analyzed allowing the identification of the most prevalent and abundant bacterial species of the salivary and dental plaque microbiota of healthy dogs. Furthermore, these analyses not only allowed to highlight the subdivision of samples into distinct recurrent bacterial compositions, but also underlined that the microbiota of the two microniches are characterized by a high abundance of bacterial species not yet characterized, underling how both bacterial ecosystems are rich reservoirs of not yet isolated and characterized species. At the same time, taxonomic differences in the microbiota of dental plaques collected from anterior or posterior teeth were observed, suggesting how the different intrinsic and extrinsic factors characterizing the microniche of the two different dental plaque locations may play a fundamental role in influencing their bacterial composition.

Furthermore, by comparing the salivary and dental plaque microbiota of healthy dogs with those from dogs affected by CGI or periodontitis, bacterial and functional biomarkers associated with periodontal diseases were identified. However, since a large part of the identified microbial biomarkers corresponded to taxa not yet isolated and characterized, the application of a culture-dependent approach aimed at the isolation of these putative novel species could help to provide more accurate information about their involvement in periodontal diseases by scrutinizing their genetic repertoire and associated metabolic functions and evaluate whether the oral pathologies can be exclusively attributable to a higher or lesser abundance of a certain species or, rather, to strain-specific genetic signature. In addition, a culture-based approach combined with the whole genome sequencing of new isolated species would help to expand the reference database allowing to obtain more accurate information regarding the composition of the oral microbiota of dogs at the species level.

Furthermore, since a reduced number of samples from dogs with chronic gingivitis and periodontitis were collected, further investigations with a larger number of samples could provide higher statistical robustness to the identification of microbial and functional biomarkers related to periodontal diseases and, at the same time, would allow the application of machine learning models to effectively predict the biomarkers of a specific clinical condition. In addition, the application of deep shotgun sequencing to saliva and dental plaque samples may be useful to achieve more accurate information about the taxonomic composition and functional features of the oral microbiota in healthy and diseased dogs, including the investigation of the presence of antibiotic resistance genes. Furthermore, since most oral disease biomarkers correspond to low abundance microbial taxa, a deep shotgun approach may help to better understand the functional potential of these species and investigate the presence of potential genetic biomarkers possibly involved in canine oral diseases. Another limitation of the current study is represented by the lack of data on saliva pH. Indeed, since it has been demonstrated that pH plays an important role in affecting the taxonomic composition of saliva (Iacopetti et al. 2017, Damian et al. 2018, Pasha et al. 2018), further studies aimed at correlating saliva pH and microbial composition may provide a better characterization of this oral microniche. However, despite these limitations, the present study provides a detailed species-level characterization of the microbial community of saliva as well as anterior and posterior dental plaques in dogs coupled with the identification of both microbial and functional biomarkers of the two most widespread canine oral diseases.

## Supplementary Material

fiae082_Supplemental_Files
