# Supplementary material for: Species-level characterization of saliva and dental plaque microbiota reveals putative bacterial and functional biomarkers of periodontal diseases in dogs
Source: FEMS Microbiol Ecol. 2024 May 23;100(6):fiae082. doi: 10.1093/femsec/fiae082 (PMC11165276; doi:10.1093/femsec/fiae082)
Supplement: fiae082_Supplemental_Files [file fiae082_supplemental_files.zip › Supplementary data_text_v3_revision.docx]

**Species-level characterization of saliva and dental plaque microbiota reveals putative bacterial and functional biomarkers of periodontal diseases in dogs**

Running title: oral microbiota in periodontal diseases in dogs

Key words: oral cavity, canine, premolars, metagenomics, shallow shotgun, chronic gingival inflammation, periodontitis

Giulia Alessandri^1^, Federico Fontana^1,2^, Leonardo Mancabelli^3,5^, Chiara Tarracchini^1^, Gabriele Andrea Lugli^1^, Chiara Argentini^1^, Giulia Longhi^1,2^, Sonia Mirjam Rizzo^1^, Laura Maria Vergna^1^, Rosaria Anzalone^2^, Alice Viappiani^2^, Francesca Turroni^1,5^, Maria Cristina Ossiprandi^4,5^, Christian Milani^1,5*^, and Marco Ventura^1,5*^

[giulia.alessandri@unipr.it](mailto:giulia.alessandri@unipr.it), [federico.fontana1@unipr.it](mailto:federico.fontana1@unipr.it), [leonardo.mancabelli@unipr.it](mailto:leonardo.mancabelli@unipr.it), [chiara.tarracchini@unipr.it](mailto:chiara.tarracchini@unipr.it), [gabrieleandrea.lugli@unipr.it](mailto:gabrieleandrea.lugli@unipr.it), [chiara.argentini@unipr.it](mailto:chiara.argentini@unipr.it), [giulia.longhi@unipr.it](mailto:giulia.longhi@unipr.it), [soniamirjam.rizzo@unipr.it](mailto:soniamirjam.rizzo@unipr.it), [lauramaria.vergna@unipr.it](mailto:lauramaria.vergna@unipr.it), [rosaria.anzalone@genprobio.com](mailto:rosaria.anzalone@genprobio.com), [alice.viappiani@genprobio.com](mailto:alice.viappiani@genprobio.com), [francesca.turroni@unipr.it](mailto:francesca.turroni@unipr.it), [mariacristina.ossiprandi@unipr.it](mailto:mariacristina.ossiprandi@unipr.it), [christian.milani@unipr.it](mailto:christian.milani@unipr.it), and [marco.ventura@unipr.it](mailto:marco.ventura@unipr.it)

^1^Laboratory of Probiogenomics, Department of Chemistry, Life Sciences, and Environmental Sustainability, University of Parma, Parma, Italy; ^2^GenProbio srl, Parma, Italy; ^3^Department of Medicine and Surgery, University of Parma, Parma, Italy; ^4^Department of Veterinary Medical Science, University of Parma, Parma, Italy; ^5^Microbiome Research Hub, University of Parma, Parma, Italy

^*^Corresponding authors

Mailing address for Christian Milani, Laboratory of Probiogenomics, Department of Chemistry, Life Sciences, and Environmental Sustainability, University of Parma, Parco Area delle Scienze 11a, 43124 Parma, Italy. Phone: ++39-521-905666. Fax: ++39-521-905604. E-mail: [christian.milani@unipr.it](mailto:christian.milani@unipr.it)

Mailing address for Marco Ventura, Laboratory of Probiogenomics, Department of Chemistry, Life Sciences, and Environmental Sustainability, University of Parma, Parco Area delle Scienze 11a, 43124 Parma, Italy. Phone: ++39-521-905666. Fax: ++39-521-905604. E-mail: [marco.ventura@unipr.it](mailto:marco.ventura@unipr.it)

**Phylum- and genus-level insight into the oral bacterial community of dogs.** To dissect the oral bacterial ecosystem of dogs, the taxonomic composition of the collected samples was explored at the phylum level. In detail, although a total of 16 different bacterial phyla were detected (Table S3), only seven dominated the oral environment of dogs (prevalence > 90%), including Bacteroidota (average relative abundance of 49.03%), Actinomycetota (18.70%), Pseudomonadota (18.45%), Bacillota (5.39%), Spirochaetota (3.26%), Campylobacterota (1.62%), and Fusobacteriota (0.86%) (Table S3). In addition, since the latter were identified both in saliva and in dental plaques, as well as in both healthy samples and samples from dogs with periodontal diseases, it can be suggested that these bacterial phyla are typical colonizers of the canine oral microbiota regardless of the oral micro-niche and clinical status.

Furthermore, to evaluate whether chronic gingival inflammation and/or periodontitis were associated with a phylum-level modulation of the bacterial composition of the oral microbiota in dogs, the taxonomic composition of samples collected from healthy dogs was compared to that obtained from samples retrieved from dogs affected by CGI or periodontitis for each sample type. In detail, none of the most representative bacterial phyla of the oral microbiota of dogs differed significantly among the three different clinical conditions for none of the considered micro-niches (Kruskal-Wallis test *p*-value > 0.05), except for Fusobacteriota whose relative abundance was significantly lower in the anterior dental plaques collected from dogs with CGI compared to healthy dogs or dogs with periodontitis (Kruskal-Wallis *p*-value of 0.022 and 0.024, respectively) (Table S4), suggesting that canine oral diseases may be associated, in general, with changes in the relative abundance of accessory bacterial phyla. In this context, for all three considered ecological oral micro-niches, the phylum Euryarchaeota showed a significantly higher abundance in dogs affected by periodontitis when compared to the healthy ones (Kruskal-Wallis test *p*-value <0.05) indicating that an increase of this taxon may be a biomarker of periodontitis (Table S3 and Table S4). At the same time, while a significant increase in the abundance of Mycoplasmatota was observed in the saliva of dogs affected by periodontitis with respect to the healthy ones (Kruskal-Wallis test *p*-value < 0.01), suggesting this microbial phylum as a biomarker of periodontitis for saliva, the abundance of the phyla Campylobacterota was significantly higher in the anterior dental plaque of healthy dogs when compared to those affected by CGI (Kruskal-Wallis test *p*-value = 0.041), indicating that this bacterial taxon may be associated with a healthy condition (Table S3 and Table S4).

Beyond the phylum-level composition, to obtain an in-depth insight into the makeup of the oral microbiota of dogs, the taxonomic profiles at the genus-level were also investigated. In this context, of the 451 bacterial genera identified, only 12 corresponded to the most abundant (average relative abundance > 1%) and prevalent (prevalence > 80%) ones, including *Porphyromonas* (average relative abundance of 36.91%), *Corynebacterium* (9.83%), *Tannerella* (4.07%), *Treponema* (3.26%), *Desulfomicrobium* (3.18%), *Actinomyces* (3.08%), *Neisseria* (2.77%), *Moraxella* (2.33%), *Capnocytophaga* (2.27%), *Bacteroides* (1.63%), *Campylobacter* (1.51%), and *Prevotella* (1.34%), confirming what was previously observed (1-5) (Table S3) In detail, since these bacterial genera were shared among the different sample types as well as among samples of different clinical conditions, it can be hypothesized that these taxa have undergone an extensive co-evolution with the host and that their presence in the canine oral cavity is independent of the ecological micro-niche as well as the clinical condition. Notably, the relative abundance of these dominant bacterial genera was not significantly different when subdividing samples by clinical conditions (Krusal-Wallis p-value > 0.05), except for the genus *Campylobacter* whose relative abundance was significantly higher in the anterior dental plaques of healthy dogs when compared to those collected from dogs with CGI (Krusal-Wallis p-value = 0.043) (Table S4). On the contrary, the other 3, 44, and 14 bacterial genera that significantly varied among clinical conditions for anterior and posterior dental plaques and saliva, respectively, corresponded to taxa with low average relative abundance, indicating that, as above described at the phylum-level, dental diseases in dogs are generally accompanied by an alteration of the less abundant genera (Table S4).

Overall, these results allowed to identify the most abundant and prevalent bacterial phyla and genera of the oral microbial ecosystem of dogs and pointed out that oral diseases in dogs are generally associated with changes in less abundant taxa rather than in most representative phyla or genera.

**References**

1. Oba PM, Carroll MQ, Alexander C, Valentine H, Somrak AJ, Keating SCJ, Sage AM, Swanson KS. 2021. Microbiota populations in supragingival plaque, subgingival plaque, and saliva habitats of adult dogs. Anim Microbiome 3:38.

2. Oba PM, Carroll MQ, Alexander C, Somrak AJ, Keating SCJ, Sage AM, Swanson KS. 2021. Dental chews positively shift the oral microbiota of adult dogs. J Anim Sci 99.

3. Ruparell A, Inui T, Staunton R, Wallis C, Deusch O, Holcombe LJ. 2020. The canine oral microbiome: variation in bacterial populations across different niches. BMC Microbiol 20:42.

4. Lisjak A, Correa Lopes B, Pilla R, Nemec A, Suchodolski JS, Tozon N. 2023. A Comparison of the Oral Microbiota in Healthy Dogs and Dogs with Oral Tumors. Animals (Basel) 13.

5. Sakarnyte L, Siugzdiniene R, Zymantiene J, Ruzauskas M. 2023. Comparison of Oral Microbial Composition and Determinants Encoding Antimicrobial Resistance in Dogs and Their Owners. Antibiotics (Basel) 12.
