# Supplementary material for: Species-level characterization of saliva and dental plaque microbiota reveals putative bacterial and functional biomarkers of periodontal diseases in dogs
Source: FEMS Microbiol Ecol. 2024 May 23;100(6):fiae082. doi: 10.1093/femsec/fiae082 (PMC11165276; doi:10.1093/femsec/fiae082)
Supplement: fiae082_Supplemental_Files [file fiae082_supplemental_files.zip › Supplementary_figure_legend.docx]

**Species-level characterization of saliva and dental plaque microbiota reveals putative bacterial and functional biomarkers of periodontal diseases in dogs**

Running title: oral microbiota in periodontal diseases in dogs

Key words: oral cavity, canine, premolars, metagenomics, shallow shotgun, chronic gingival inflammation, periodontitis

Giulia Alessandri1, Federico Fontana1,2, Leonardo Mancabelli3,5, Chiara Tarracchini1, Gabriele Andrea Lugli1, Chiara Argentini1, Giulia Longhi1,2, Sonia Mirjam Rizzo1, Laura Maria Vergna1, Rosaria Anzalone2, Alice Viappiani2, Francesca Turroni1,5, Maria Cristina Ossiprandi4,5, Christian Milani1,5*, and Marco Ventura1,5*

[giulia.alessandri@unipr.it](mailto:giulia.alessandri@unipr.it), [federico.fontana1@unipr.it,](mailto:federico.fontana1@unipr.it) [leonardo.mancabelli@unipr.it](mailto:leonardo.mancabelli@unipr.it),

[chiara.tarracchini@unipr.it,](mailto:chiara.tarracchini@unipr.it) [gabrieleandrea.lugli@unipr.it](mailto:gabrieleandrea.lugli@unipr.it), [chiara.argentini@unipr.it,](mailto:chiara.argentini@unipr.it)

[giulia.longhi@unipr.it](mailto:giulia.longhi@unipr.it), [soniamirjam.rizzo@unipr.it](mailto:soniamirjam.rizzo@unipr.it), [lauramaria.vergna@unipr.it](mailto:lauramaria.vergna@unipr.it),

[rosaria.anzalone@genprobio.com,](mailto:rosaria.anzalone@genprobio.com) [alice.viappiani@genprobio.com,](mailto:alice.viappiani@genprobio.com) [francesca.turroni@unipr.it,](mailto:francesca.turroni@unipr.it)

[mariacristina.ossiprandi@unipr.it,](mailto:mariacristina.ossiprandi@unipr.it) [christian.milani@unipr.it,](mailto:christian.milani@unipr.it) and [marco.ventura@unipr.it](mailto:marco.ventura@unipr.it)

1Laboratory of Probiogenomics, Department of Chemistry, Life Sciences, and Environmental Sustainability, University of Parma, Parma, Italy; 2GenProbio srl, Parma, Italy; 3Department of Medicine and Surgery, University of Parma, Parma, Italy; 4Department of Veterinary Medical Science, University of Parma, Parma, Italy; 5Microbiome Research Hub, University of Parma, Parma, Italy

*Corresponding authors

Mailing address for Marco Ventura, Laboratory of Probiogenomics, Department of Chemistry, Life Sciences, and Environmental Sustainability, University of Parma, Parco Area delle Scienze

11a, 43124 Parma, Italy. Phone: ++39-521-905666. Fax: ++39-521-905604. E-mail:

[marco.ventura@unipr.it](mailto:marco.ventura@unipr.it)

Mailing address for Christian Milani, Laboratory of Probiogenomics, Department of Chemistry, Life Sciences, and Environmental Sustainability, University of Parma, Parco Area delle Scienze

11a, 43124 Parma, Italy. Phone: ++39-521-905666. Fax: ++39-521-905604. E-mail:

[christian.milani@unipr.it](mailto:christian.milani@unipr.it)

**Supplementary figure legends**

**Figure S1:** Graphs represent Silohouette index, obtained through the heuristic Silhouette method to determine the number of clusters in a data set, for saliva (a), anterior (b), and posterior (c) dental plaque in healthy dogs.

**Figure S2:** Identification of distinct compositional patterns of the saliva and dental plaque microbiota in healthy dogs. Bar charts report the average relative abundance of each bacterial species characterizing each identified Community State Type (CST) in saliva (a), anterior (b), and posterior (c) dental plaque samples. The cladogram on the top of each bar chart corresponds to the hierarchical clustering of the samples based on their species-level taxonomic profiles. Only dominant bacterial species (average relative abundance >10%) for each CST are shown.
