# Supplementary figures and images for: Species-level characterization of saliva and dental plaque microbiota reveals putative bacterial and functional biomarkers of periodontal diseases in dogs
Source: FEMS Microbiol Ecol. 2024 May 23;100(6):fiae082. doi: 10.1093/femsec/fiae082 (PMC11165276; doi:10.1093/femsec/fiae082)

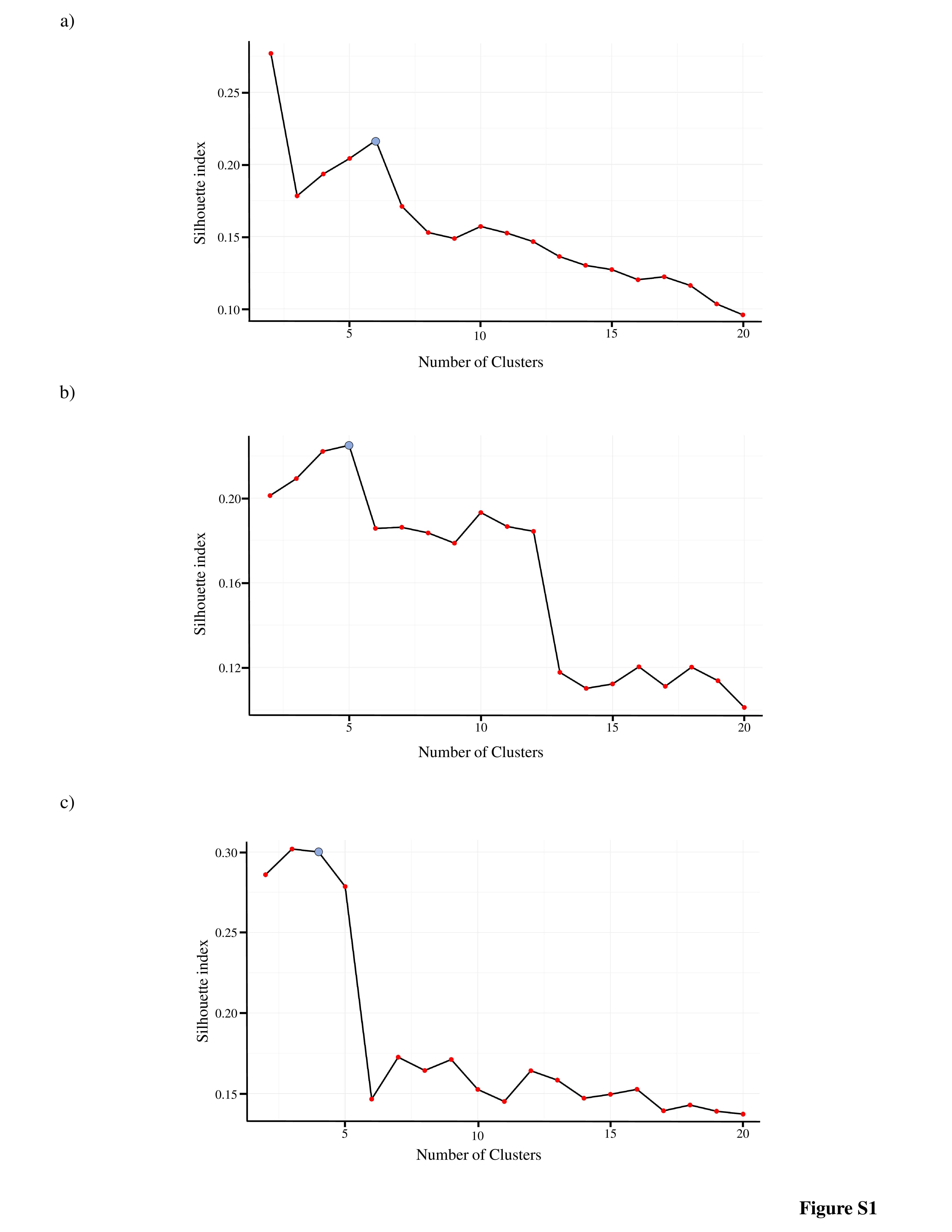

Supplement: fiae082_Supplemental_Files [file fiae082_supplemental_files.zip › supp data FigureS1_revision.tif]

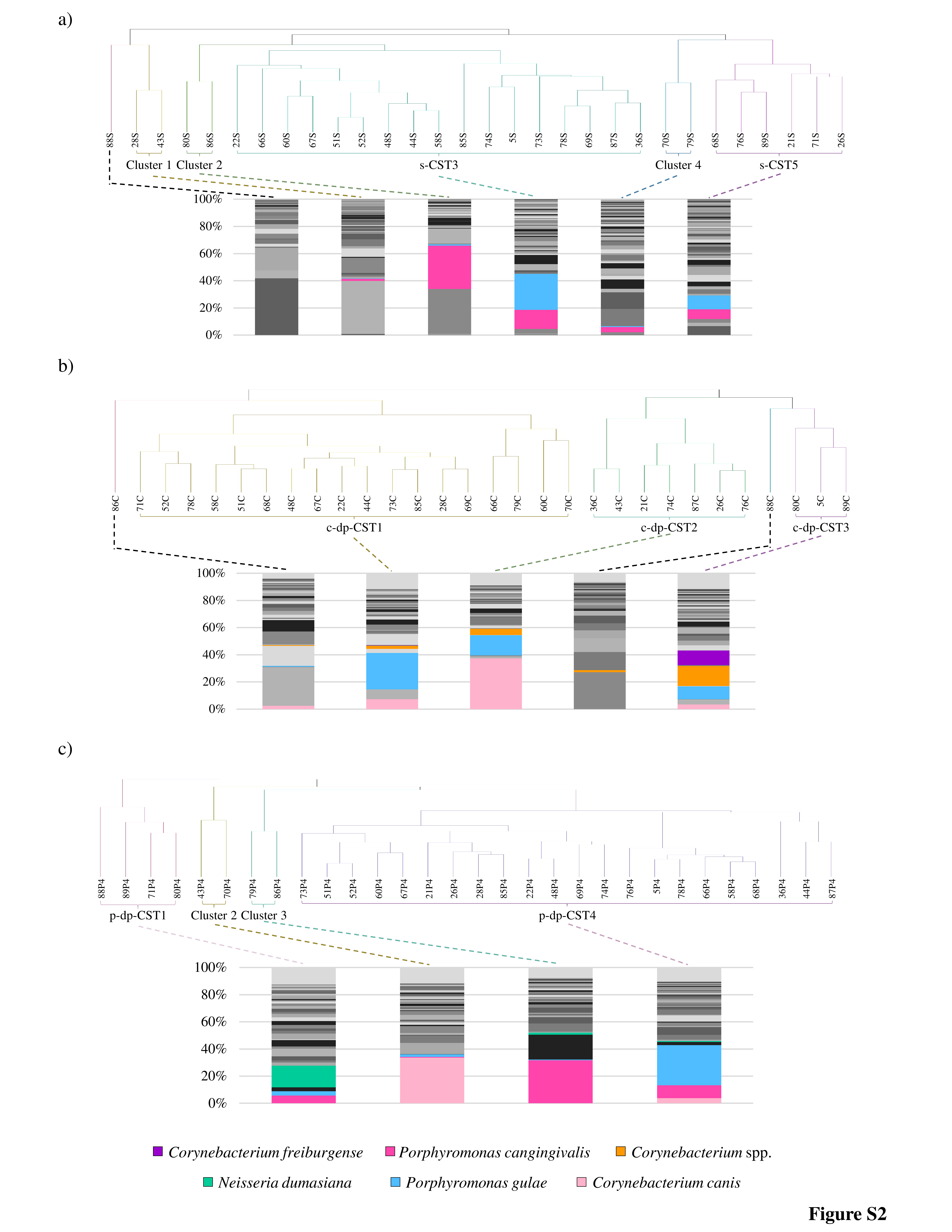

Supplement: fiae082_Supplemental_Files [file fiae082_supplemental_files.zip › supp data FigureS2_revision_v2.tif]
